# Older Age Increases the Amplitude of Muscle Stretch-Induced Cortical Beta-Band Suppression But Does not Affect Rebound Strength

**DOI:** 10.3389/fnagi.2020.00117

**Published:** 2020-05-19

**Authors:** Simon Walker, Simo Monto, Jarmo M. Piirainen, Janne Avela, Ina M. Tarkka, Tiina M. Parviainen, Harri Piitulainen

**Affiliations:** ^1^NeuroMuscular Research Center, Faculty of Sport and Health Sciences, University of Jyväskylä, Jyväskylä, Finland; ^2^Department of Psychology, Centre for Interdisciplinary Brain Research, University of Jyväskylä, Jyväskylä, Finland; ^3^Faculty of Sport and Health Sciences and Centre for Interdisciplinary Brain Research, University of Jyväskylä, Jyväskylä, Finland; ^4^Department of Neuroscience and Biomedical Engineering, Aalto University School of Science, Espoo, Finland

**Keywords:** event-related desynchronization (ERD), sensorimotor, lower limbs, proprioception, somatosensory processing, MEG

## Abstract

Healthy aging is associated with deterioration of the sensorimotor system, which impairs balance and somatosensation. However, the exact age-related changes in the cortical processing of sensorimotor integration are unclear. This study investigated primary sensorimotor cortex (SM1) oscillations in the 15–30 Hz beta band at rest and following (involuntary) rapid stretches to the triceps surae muscles (i.e., proprioceptive stimulation) of young and older adults. A custom-built, magnetoencephalography (MEG)-compatible device was used to deliver rapid (190°·s^−1^) ankle rotations as subjects sat passively in a magnetically-shielded room while MEG recorded their cortical signals. Eleven young (age 25 ± 3 years) and 12 older (age 70 ± 3 years) adults matched for physical activity level demonstrated clear 15–30 Hz beta band suppression and rebound in response to the stretches. A sub-sample (10 young and nine older) were tested for dynamic balance control on a sliding platform. Older adults had greater cortical beta power pre-stretch (e.g., right leg: 4.0 ± 1.6 fT vs. 5.6 ± 1.7 fT, *P* = 0.044) and, subsequently, greater normalized movement-related cortical beta suppression post-proprioceptive stimulation (e.g., right leg: −5.8 ± 1.3 vs. −7.6 ± 1.7, *P* = 0.01) than young adults. Furthermore, poorer balance was associated with stronger cortical beta suppression following proprioceptive stimulation (*r* = −0.478, *P* = 0.038, *n* = 19). These results provide further support that cortical processing of proprioception is hindered in older adults, potentially (adversely) influencing sensorimotor integration. This was demonstrated by the impairment of prompt motor action control, i.e., regaining perturbed balance. Finally, SM1 cortex beta suppression to a proprioceptive stimulus seems to indicate poorer sensorimotor functioning in older adults.

## Introduction

Aging is associated with deterioration in maximum force production independent of muscle size (Ojanen et al., 2007), worse rapid force production (Skelton et al., 1994), decreased low-force contraction steadiness (Galganski et al., 1993), and poorer dynamic balance control during perturbations (Piirainen et al., 2013). Proprioceptors are located within muscles and joints, and they sense movement/limb position and forces. Proprioception is impaired with aging (Kaplan et al., 1985) and this is considered to be one of the main contributors to loss of balance in older adults (Lord and Ward, 1994). Proprioception is not solely a peripheral phenomenon, and recently we have observed impaired proprioceptive processing within the primary sensorimotor cortex (SM1; Piitulainen et al., 2018). These age-related maladaptations suggest altered neuronal motor control, but the exact causes of the modified neuronal control in various conditions (e.g., during perturbed balance control) remain elusive.

Stretch-reflexes are important in overcoming perturbations during standing balance. The post-stretch short-latency activity of the muscle purportedly represents a monosynaptic spinal pathway (i.e., Ia muscle spindle afferents excite the α-motoneurons of the spinal cord). Once proprioceptive afference from stretching reaches the cortex (Dietz et al., 1985), the modulation of oscillating neuronal activity occurs (van Boxtel, 1976). Thereafter, a cortically-driven response reaches the muscle (Dietz et al., 1985), hence, the post-stretch long-latency activity reflects a polysynaptic cortical pathway (Taube et al., 2006; Shemmell et al., 2010; Piirainen et al., 2013). Based on transcranial magnetic stimulation findings, it has been suggested that altered cortical control modifies (spinal-level) reflexes during upright standing, particularly in older adults (Baudry et al., 2015), which may lead to greater cortical influence during standing balance in older adults (Baudry, 2016). This may reflect compensation to overall age-related impairment of the sensorimotor system both at cortical and spinal levels that may lead to greater demand on cortical influence during motor actions. Indeed, our findings of altered cortical proprioceptive processing was related to standing balance performance (Piitulainen et al., 2018).

Using electroencephalography, Ozdemir et al. (2018) observed earlier and greater N1 responses over the central electrode sites in young compared to older adults during perturbed standing balance. It has been proposed that N1 responses represent the cortical processing of somatosensory information (Dietz et al., 1985). However, cortical-evoked activation is only one part of the overall somatosensory activity during standing balance; induced modulations to rhythmic cortical activity (i.e., oscillations) also occur and could have functional relevance in efficient motor control.

Cortical oscillations have been suggested to be associated with efferent motor control as well as processing of afferent somatosensory input, with a particular interest on the beta band (15–30 Hz) during voluntary motor tasks. Clear beta power suppression [also known as event-related desynchronization (ERD)] occurs approximately at the onset of voluntary actions and shortly after somatosensory stimuli, and is followed by an accentuated recovery or rebound (also known as event-related resynchronization) post-movement (Salmelin and Hari, 1994; Stancák and Pfurtscheller, 1995; Parkkonen et al., 2015). Beta suppression reflects activation of the SM1 cortex and is likely related to early processing of somatosensory afference. Indeed, slow passive rotations of the ankle joint led to beta suppression and rebound in both young and older subjects, but suppression occurring at a shorter latency was accompanied by a faster response time in a button press task (Toledo et al., 2016).

Recently, understanding of the factors influencing beta suppression and rebound amplitude has increased with studies showing that certain factors reduce the amplitude of the rebound, such as childhood (Gaetz et al., 2010), demyelination (Arpin et al., 2017), ischemia (Cassim et al., 2001), and stroke (Laaksonen et al., 2012). It has been speculated that the strength of beta rebound reflects the active inhibition or reduced excitability of the SM1 cortex (Pfurtscheller, 1992; Salmelin et al., 1995) and is a GABA-mediated process (Muthukumaraswamy et al., 2013). Interestingly, weaker rebound has been linked with worse force accuracy (Arpin et al., 2017) and impaired hand dexterity (Laaksonen et al., 2012). Additionally, stronger rebound is associated with better recovery after stroke (Parkkonen et al., 2017). Together, these findings indicate that beta rebound could serve as a cortical signature of sensorimotor function.

The neuronal mechanisms governing beta suppression and rebound are distinct. Good examples of this are that Diazepam administration (Hall et al., 2011) increased suppression but not rebound strength, and that modulation of the task (different observation/movement) during peripheral nerve stimulation affected rebound but not suppression (Muthukumaraswamy and Johnson, 2004). Further support for two distinct generators of beta suppression and rebound have been provided by studies that have identified different sources of these events (Jurkiewicz et al., 2006; Muthukumaraswamy et al., 2013), with suppression located in the primary somatosensory cortex (SM1) and rebound located in the primary motor cortex (M1). Baseline beta power also varies over time; it is weaker after fatiguing contractions (Tecchio et al., 2006) and stronger with increasing age (Rossiter et al., 2014) and following Diazepam administration (Hall et al., 2011).

To our knowledge, no study has investigated the effect of age on resting and proprioceptive stimulus-related beta band modulations to (involuntary) rapid stretching of the triceps surae muscles. Comparing beta band dynamics between young and older adults could elucidate the cortical mechanisms underlying aging and motor control of functionally relevant actions, such as maintaining standing balance. Hence, the purpose of the present study was to determine whether: (1) 15–30 Hz (beta) band modulation followed proprioceptive stimulation; and (2) beta power at rest differs between healthy older and young adults. In addition, we aimed to determine whether cortical beta band features are related to functionally relevant dynamic balance performance.

## Materials and Methods

### Subjects

Thirty-five individuals were recruited as part of an investigation into the effects of age on static and dynamic performance through advertisements in the local area (Walker et al., 2019). As part of the second arm of that study, subjects were screened by a standardized questionnaire that has been used in our previous studies. Inclusion criteria was as follows: aged between 18 and 35 or 65 and 75 years, no history of neurological or movement disorder, no metal implants or dental corrections, no use of medication known to affect the central nervous system or endocrine systems, no use of walking aids, height below 182 cm (due to MEG chair/device restrictions), and low level of physical activity (i.e., not meeting the recommended physical activity requirements). Since habitual physical activity reduces in-line with age, it was important to select only individuals with consistently low physical activity to evaluate the effects of aging specifically. Eligible subjects provided their written informed consent after being fully informed of the procedures and potential harms. The study was approved by the ethics committee of the University of Jyväskylä, Finland, and conducted according to the Declaration of Helsinki. Twenty-five subjects were measured in the present study, but one young and one older subject were excluded from further analyses due to bad signal quality. Therefore, 11 young (three men and eight women, age 25 ± 3 years, height 171 ± 9 cm, weight 67 ± 10 kg, BMI 23 ± 4) and 12 older (seven men and five women, age 70 ± 3 years, height 168 ± 9 cm, weight 76 ± 11 kg, BMI 27 ± 3) subjects were entered into the final analyses. All 23 subjects reported their preferred kicking leg to be the right leg and thus it was considered to be the dominant leg.

### MEG Experimental Procedures

Subjects attended a test session in the MEG lab of the Centre for Interdisciplinary Brain Research (CIBR) of the University of Jyväskylä. MEG signals were recorded in a magnetically-shielded room (Magnetical Shielding Cabin, VACOSHIELD, Vacuumschmelze GmbH and Co. KG, Hanau, Germany) with a 306-channel whole-scalp neuromagnetometer (Elekta Neuromagr TRIUX™, Elekta Oy, Helsinki, Finland). Signals were recorded using a bandpass of 0.1–330 Hz and the sampling rate was 1,000 Hz. The individual’s head position inside the MEG helmet was continuously monitored by a feeding current to five head-tracking coils (continuous head position indicator coils, cHPIs). The coils were attached to the scalp prior to measurement and their locations were determined with respect to anatomical fiducials with an electromagnetic tracker (Fastrak, Polhemus, Colchester, VT, USA). Eye movements and blinks were tracked by electrooculography (EOG). The subjects’ initial head position was recorded and compared between groups. There were no between-group differences in any of the three coordinates, with the average difference in the vertical direction being ~0.6 mm (e.g., Z-coordinate; Young: −2.3 ± 2.4 mm vs. Older: −2.9 ± 4.1 mm, *P* = 0.639, with respect to the default coordinate system of the MEG device).

**Figure 1:**
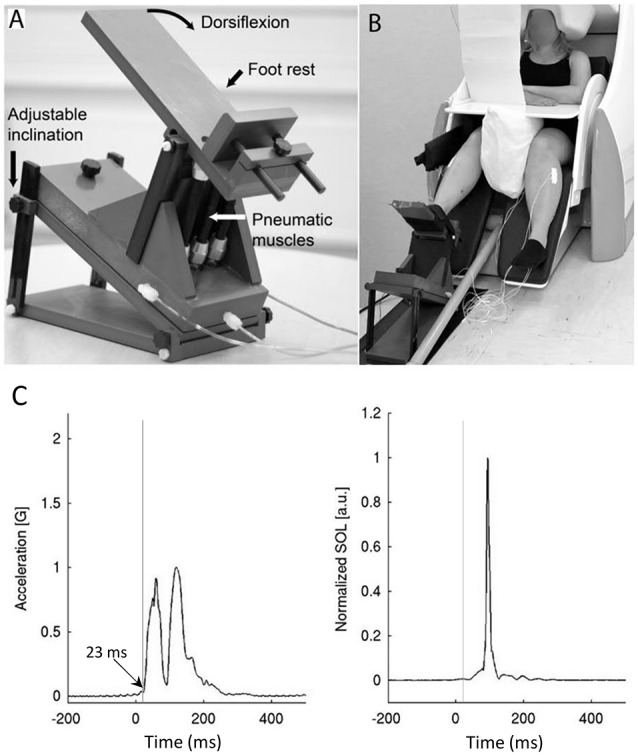
The pneumatic movement actuator used to rotate the foot about the ankle (**A**—modified from Piitulainen et al., 2018) and the experimental set-up with subject seated in the test position **(B)**. There was a consistent delay of 23 ms between the trigger and actual movement, but the rapid movements induced stretch-reflexes in the plantarflexor muscles as shown in the rectified images **(C)**.

A custom-made MEG-compatible movement actuator (Aalto NeuroImaging, Aalto University, Espoo, Finland, see details from Piitulainen et al., 2018) was used to rotate the foot about the ankle with the subjects sitting in a completely relaxed state. The pneumatic system used air pressure (1–7 bar) to contract three artificial muscles (DMSP-10-100 AM-CM, diameter 10 mm, length of the contracting part 100 mm; Festo AG and Co., Esslingen, Germany) attached to the heel part of the footplate, thus rotating the footplate upon injection (dorsiflexion) and ejection (plantarflexion) of air (Figure 1A). Air pressure was regulated by a solenoid valve (SY5220-6LOU-01F-Q, SMC Corporation, Tokyo, Japan) located outside of the shielded room, which was controlled by computer-generated trigger pulses.

During MEG recordings, the subjects were sitting with their eyes open with the foot of the stimulated leg strapped to the movement actuator, which was positioned on a non-slip mat on the floor of the shielded room. The non-stimulated leg was resting on the leg support of the chair (Figure 1B). Earplugs were used and Brownian noise was played in the background *via* a flat-panel speaker (Panphonics 60 × 60 SSHP, Tampere, Finland) to block concomitant low auditory noise that arose from the movement actuator. The suitability of the masking noise was checked prior to recordings and, if necessary, the volume was increased. Subjects were prevented from seeing the moving leg with a white sheet, taped vertically to the MEG gantry. Subjects were instructed to fixate on a black dot on the wall of the magnetically shielded room, 3 m in front of them. A pillow was placed between the legs to secure the resting position and the hands were placed on the table to minimize any additional movement.

Rapid dorsiflexion movements (movement range ~6.6°, peak angular velocity 190°·s^−1^) inducing stretch-reflexes were generated for the ankle joint at random intervals after the foot pedal had returned to the start position (range 4-8 s, mean 6 s) for each leg separately. Additionally, the foot pedal of the device remained stationary in the dorsiflexed position for 2 s after movement before returning to the start position. A total of 75 dorsiflexions were induced for each leg during the recordings. The mechanical stretching order of the legs was randomized for each individual. The subjects remained completely relaxed during the recordings and were instructed not to contract their muscles at any point during the mechanical stretching. Accelerometer data from a 3-axis accelerometer (ADXL335 iMEMS Accelerometer, Analog Devices Inc., Norwood, MA, USA) attached on the skin over the metatarsal bone showed a consistent 23 ms pneumatic delay between the onset of the trigger signal and onset of the actual ankle rotation (Figure 1C). This delay has been subtracted from all data.

### Electromyography

The electrical activity of the muscles during the measurements was recorded by electromyography (EMG) electrodes (Neuroline 720, Ambu A/S, Denmark) in bipolar arrangement (pre-gelled and adhesive Ag/AgCl electrodes, 5 mm diameter, 20 mm inter-electrode distance). EMG activity was measured from the m. soleus, m. gastrocnemius, and m. tibialis anterior of both legs following SENIAM guidelines (Hermens et al., 1999). A ground electrode was placed on the clavicle. EMG-activity was measured simultaneously with MEG (Elekta Neuromag^®^ TRIUX™, Elekta Oy, Helsinki, Finland; 100 MΩ input impedance, <1 mV rms baseline noise) using the same bandpass filter (0.1–330 Hz) and sampling rate (1,000 Hz).

### Dynamic Balance Tests

In a sub-set of subjects (young: *n* = 10, older *n* = 9), dynamic balance performance to perturbations induced by a horizontally sliding force platform were measured. This platform is known to induce stretch-reflex responses of the triceps surae muscles (Piirainen et al., 2013). Subjects underwent a familiarization session to become accustomed to the test procedures 3 days prior to testing. During testing, subjects stood with feet hip-width apart and hands positioned together (resting) in front of the body. The platform slid forwards (six trials) or backwards (six trials) every 8–12 s in a randomized order so that the subjects could not anticipate the stretches. The platform was programmed to slide at a maximum acceleration of 2.9 m·s^−2^, maximum velocity of 24 cm·s^−1^, and total displacement of 30 cm. A black cross was fixed on the wall 3 m from the subject at eye level to stabilize the subject’s visual focus during the measurements. Peak anterior-posterior center-of-pressure (CoP) displacement and velocity were analyzed. Only trials where the platform slid backwards were taken into the analyses, as this engages the triceps surae muscles, and the results of the six trials were averaged.

### MEG Data Processing

Continuous MEG data were first preprocessed off-line using temporal signal-space-separation with head movement compensation to suppress external interferences and to correct for head movements during recording (Taulu and Simola, 2006). The MEG, EMG, and acceleration signals were band-pass filtered offline at 0.4–195 Hz.

MEG data was visually inspected, and any remaining bad channels were removed. All further analyses were performed using MNE Python software version 2.7.15. Any trial that was contaminated by artifacts was also removed. A bandpass filter of 1–40 Hz was applied to the raw data. Independent components analysis (FastICA, Hyvärinen and Oja, 2000) combined with automatic component selection was run to automatically detect and manually confirm artifacts derived from eye movement and/or cardiac signals. All data was manually checked for other artifacts, and those epochs were removed if necessary. Thereafter, epochs from −0.5 s to 2.0 s were created with respect to the trigger onset (0 s) and MEG signals were averaged across all epochs (72 ± 2 averages were obtained for each subject—there were no differences in the number of trials accepted to analyses between age-groups).

**Figure 2:**
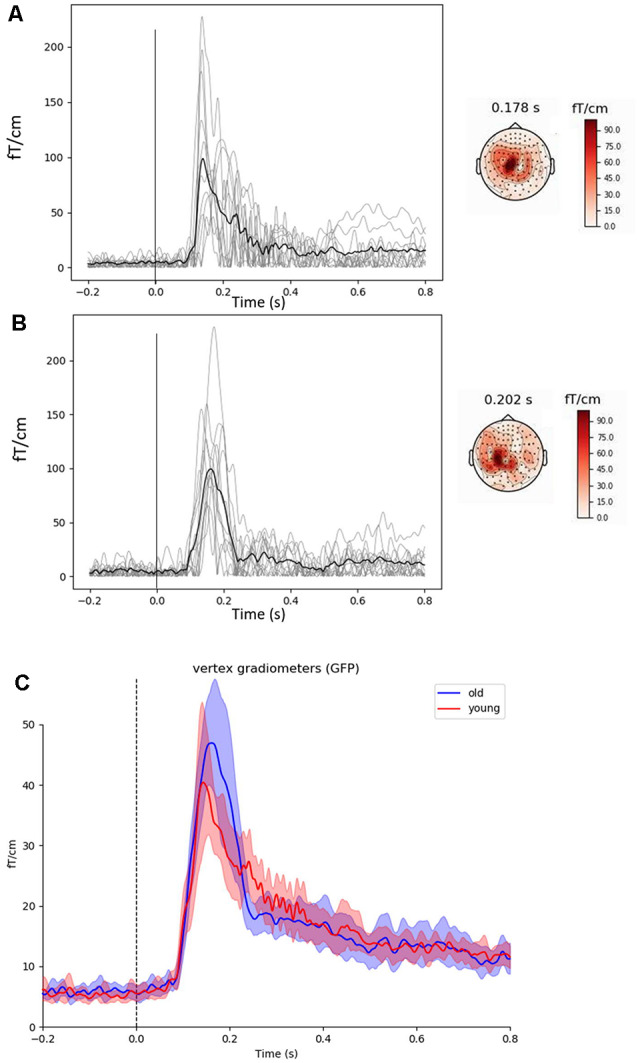
Evoked cortical fields and representative topographies induced by proprioceptive stimuli observed in the vertex gradiometer showing the highest rebound in each subject (light gray lines) along with the group mean (black line) in young **(A)** and older **(B)** subjects. Grand averages in young (red line) and older (blue line) subjects for the global field power of the 50 vertex gradiometers with 95% confidence intervals are shown in **(C)**. The main responses are located within the sensorimotor areas. The vertical line at 0 s represents movement onset. The 23 ms delay has been removed.

**Figure 3:**
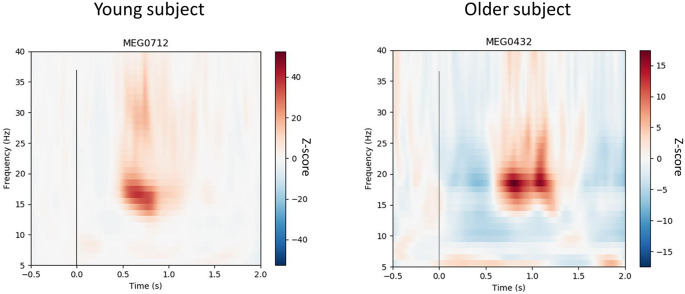
Time-frequency resolutions over 15–35 Hz normalized to pre-proprioceptive stimuli baseline (−0.5 to 0 s) from the dominant (i.e., right) leg of a representative young and older subject. The vertex gradiometer with the highest rebound for each individual was selected and shows suppression and rebound after the movement. The vertical line at 0 s represents movement onset. The 23 ms delay has been removed. The figure shows that the 15–35 Hz response to the rapid, involuntary stretching has dissipated prior to the subsequent stretch.

Fifty gradiometers from the vertex channels were isolated in order to assess proprioceptive stimuli evoked fields (Figure 2), as well as maximum and minimum beta power. Evoked responses were removed from the data prior to time-frequency analyses. Baseline was defined as −0.5 to 0 s, with which to normalize changes in frequency power following proprioceptive stimulation. Time-frequency representation (TFR) plots (Figure 3) were created and visually inspected to confirm a clear 15–30 Hz response using z-score normalization computed from baseline data, prior to performing Hilbert enveloped temporal spectral evolution analyses. The single channel showing the greatest post-proprioceptive stimulation z-score (i.e., peak beta rebound) was selected from these 50 gradiometers over the vertex region for each subject to be entered into temporal spectral evolution analyses. Similarly, peak beta suppression was taken from the single channel showing the lowest z-score from the 50 vertex gradiometers. These channels were assessed for peak amplitude, latency, and dominant frequency. Additionally, the dominant beta frequency at rest was assessed from 90 s of quiet sitting using standard power spectral density methods. These values were then assessed statistically (see procedures below). Beta power during baseline (−0.5 to 0 s) for the peak beta rebound channel was quantified to represent baseline beta power between groups (Heinrichs-Graham and Wilson, 2016).

Similar results were obtained from both the right and left leg in this study, therefore, reporting of results is restricted to the right (dominant) leg for brevity.

### Statistical Methods

Data are presented as means ± standard deviations unless otherwise stated. Normality was assessed by the Kolmogorov–Smirnov test. Independent *t*-tests were used to determine between-group differences in beta power during baseline, in baseline-normalized temporal spectral evolution analyses, and in CoP variables. Pearson’s product correlation analyses were used to assess relationships between beta band and CoP variables for the entire group. Significance was set at *P* = 0.05. All procedures were performed by SPSS software version 24 (IBM, New York, NY, USA).

## Results

### Evoked Cortical Responses to the Stretches

Figures 2A,B show the proprioceptive stimuli evoked fields from the gradiometer showing the highest rebound in each young and older subject, along with the grand-averaged global field power for the 50 vertex gradiometers of interest (Figure 2C). The peak of the evoked field from the 50 vertex gradiometers was of a similar amplitude between young and older subjects (Young: 41 ± 13 fT·cm^−1^ vs. Older: 47 ± 11 fT·cm^−1^, *P* = 0.370). Also, the latencies of the response peak were similar between the age-groups (Young: 132 ± 18 vs. Older: 156 ± 24 ms, *P* = 0.269).

### Beta Band Modulations to the Stretches

**Figure 4:**
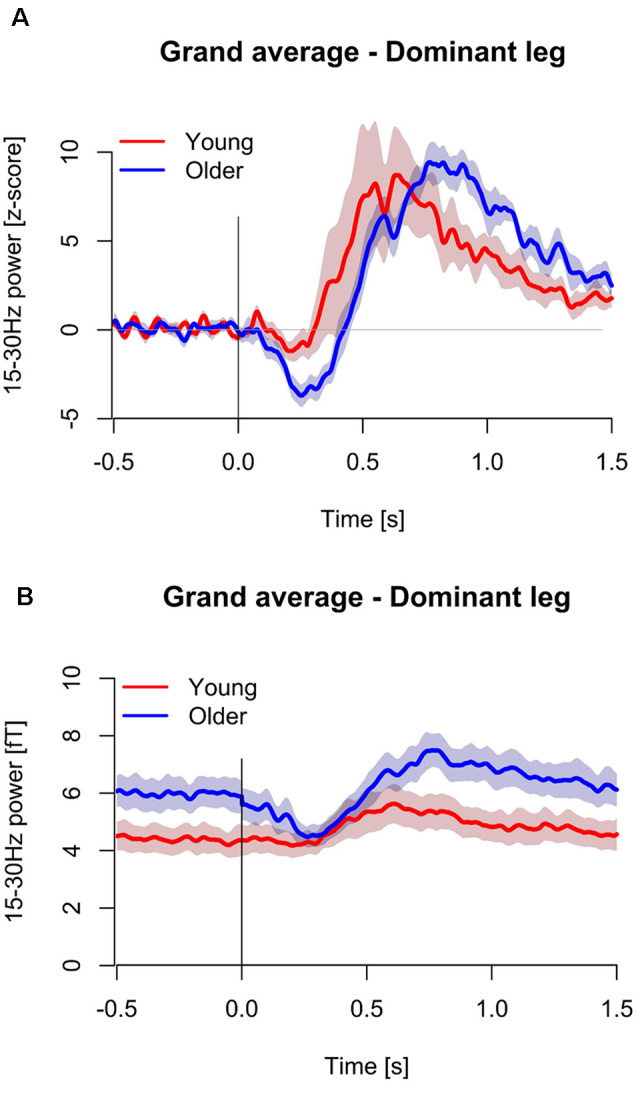
Temporal spectral evolutions for 15–30 Hz (beta) band in the young (*n* = 11, red color) and older (*n* = 12, blue color) for the dominant (i.e., right) leg. Data is presented normalized to baseline based on z-score transformations **(A)**, and as non-baseline normalized units **(B)**. Standard error regions about the mean have been shaded. The vertical line at 0 s represents movement onset. The 23 ms delay has been removed.

Figure 4A shows the group averaged temporal spectral evolution plots for the Z-score normalized baseline and 15–30 Hz (beta) band responses following proprioceptive stimulation. Peak beta suppression post-proprioceptive stimulation was significantly stronger in older subjects compared to young subjects (Young: −5.8 ± 1.3 vs. Older: −7.6 ± 1.7, *P* = 0.010). No significant differences were observed in the peak beta rebound post-proprioceptive stimulation (Young: 13.0 ± 12.1 vs. Older: 13.0 ± 8.8, *P* = 0.999) nor in the latencies for peak suppression (Young: 339 ± 120 vs. Older: 270 ± 65 ms, *P* = 0.121) or rebound (Young: 660 ± 134 vs. Older: 746 ± 146 ms, *P* = 0.079). No between-group differences were observed in the peak frequency of suppression (Young: 22.9 ± 3.5 Hz vs. Older: 25.2 ± 3.4 Hz, *P* = 0.603) or rebound (Young: 22.3 ± 5.9 Hz vs. Older: 21.1 ± 4.8 Hz, *P* = 0.135) after proprioceptive stimulation, nor during resting conditions (Young: 20.6 ± 2.3 Hz vs. Older: 21.2 ± 2.4 Hz, *P* = 0.533).

### Beta Power at Rest

Absolute beta power during the baseline period (pre-stretching) was significantly stronger in the older subjects compared to the young subjects (Young: 4.0 ± 1.6 fT vs. Older: 5.6 ± 1.7 fT, *P* = 0.044, Figure 4B). After the stimulation, the suppression in beta power in older subjects resulted in similar beta power (fT) compared to young subjects over ~750 ms (Figure 4B).

### Dynamic Standing Balance Performance

Velocity of CoP displacement differed significantly between groups (Young: 341 ± 52 vs. Older: 410 ± 81 mm·s^−1^, *P* = 0.037), however, CoP displacement did not differ significantly between groups (Young: 130 ± 10 vs. Older: 138 ± 29 mm, *P* = 0.393).

### Correlation Analyses

**Figure 5:**
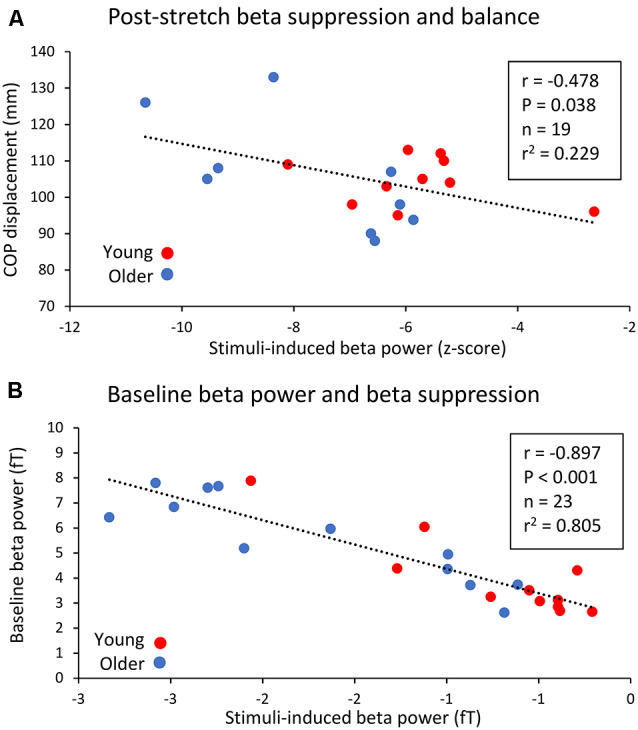
Relationship between peak 15–30 Hz (beta) band suppression post-proprioceptive stimulation in the right leg and CoP displacement during the dynamic balance tests **(A)**, as well as the relationship between baseline beta power and post-proprioceptive stimulation non-normalized suppression. **(B)** Young subjects are represented by red circles while older subjects are represented by blue circles.

CoP displacement from the perturbations was inversely related to the right leg following proprioceptive stimulation 15–30 Hz suppression (*r* = −0.478, *P* = 0.038, *n* = 19, Figure 5A). i.e., worse balance performance was associated with stronger beta suppression.

Baseline (−0.5 to 0 s) beta-band power was also inversely related to stimuli-induced beta suppression. A significant, negative relationship between baseline beta power and the peak change in beta power (fT) following right leg proprioceptive stimuli was observed (*r* = −897, *P* < 0.001, *n* = 23, Figure 5B).

## Discussion

The present study investigated the age-related differences in sensorimotor cortex oscillations in the 15–30 Hz beta band at rest and following rapid involuntary stretches (i.e., proprioceptive stimulation) to the triceps surae muscles in adults. We observed a stronger 15–30 Hz (beta) band suppression in response to proprioceptive stimulation in healthy older than young adults. Also, baseline beta power was stronger in older compared to young adults. The amplitude of the beta rebound following proprioceptive stimulation did not differ between the age-groups. Correlation analyses indicated that stronger beta suppression may be associated with an individual’s ability to recover standing balance after perturbation.

### Evoked Cortical Responses to the Proprioceptive Stimulation

When using MEG, the cortical responses to passive movement stimuli reflect primarily proprioceptive processing in the SM1 cortex with a negligible effect from cutaneous afference (Piitulainen et al., 2013; Bourguignon et al., 2015). At the spinal level, the same proprioceptive stimuli leads to muscle responses at several latencies. Proprioceptive stimulation (i.e., involuntary evoked movements) leads to muscle responses at several latencies. The long-latency response has been shown to have a cortical influence (Dietz et al., 1985; Schieppati and Nardone, 1997; Taube et al., 2006) and proprioceptive afference to the cortex has been shown to modulate alpha (~10 Hz) oscillations (van Boxtel, 1976). Furthermore, kinematics of evoked ankle rotations are strongly coupled to SM1 cortex activity (Piitulainen et al., 2018). Therefore, it appears that the cortex has an important role in sensorimotor processing related to maintaining standing balance (Baudry, 2016). In response to a perturbed stance, Ozdemir et al. (2018) observed delayed latencies for P1 (purported to be initial processing of sensory information) and N1 (purported to be higher order cortical processing) responses in older (~115 and ~219 ms, respectively) than younger (~81 and ~167 ms, respectively) adults. The present study did not observe between-group differences in either latency or amplitude on the cortical stretch evoked fields. There are two likely explanations for the disparity of findings between our study and that of Ozdemir et al. (2018). First, the older subjects in the present study were approximately 10 years younger (~70 years vs. ~80 years) than in the Ozdemir et al. (2018) study and had perhaps undergone less prominent decline in their sensorimotor system functions. Second, it is possible that the isolated single-joint plantarflexor stretches in a seated passive condition used in the present study do not sufficiently mimic the conditions of an active standing balance task. Active standing encompasses the active neural drive both to extrafusal and intrafusal (sensitizing the muscle spindles) muscle fibers and other somatosensory afference to the spinal level that are minimal or absent in the passive conditions.

### Beta Band Modulations to the Stretches

Despite the lack of age-related differences in evoked fields, stronger beta suppression was observed in the older adults in response to the ankle stretches in the present study. There were no differences in the latencies or durations of the beta suppression between the age-groups in the present study. While the latency reflects sensorimotor processing, the duration of suppression is largely regulated by the duration of contraction (Stancák and Pfurtscheller, 1995), which was a standardized brief stretch in the present study. The amplitude of beta rebound depends upon the somatosensory stimulation type or method (e.g., tactile vs. passive movement), but this does not influence the level of suppression (Parkkonen et al., 2015). Beta suppression reflects activation of the SM1 cortex (Jurkiewicz et al., 2006) and is likely related to early processing of somatosensory afference. Therefore, a stronger suppression in older adults could represent a greater (and/or more inefficient) neuronal population involved in the processing of the proprioceptive afference with age.

It has been proposed that stronger resting beta power may be linked to inhibition of the SM1 cortex and thus hinder the initiation and maintaining of motor actions (Rossiter et al., 2014; Heinrichs-Graham and Wilson, 2016). This suggestion is based on greater baseline beta power being related to stronger beta suppression during voluntary contractions. Stronger beta suppression was also accompanied by slower motor task completion (Heinrichs-Graham and Wilson, 2016). The present study’s results are in-line with these findings and are the first to investigate this phenomenon with passive lower-limb stretches. During fatigue studies, lower pre-contraction beta power was accompanied by weaker beta suppression during lower arm contractions (Tecchio et al., 2006; Fry et al., 2017). Therefore, future studies should account for baseline beta power when reporting beta suppression not solely reporting baseline-normalized values, which has not been the norm previously (e.g., Pfurtscheller et al., 2005; Proudfoot et al., 2017).

There were no age-related differences in beta rebound observed in the present study. Blunted beta rebound in response to voluntary and involuntary contractions/stretches has been observed in multiple sclerosis patients (Arpin et al., 2017), following stroke (Parkkonen et al., 2017), in amyotrophic lateral sclerosis patients (Proudfoot et al., 2017), and during ischemia (Cassim et al., 2001). Therefore, reduced beta rebound seems to be symptomatic of a dysfunctional somatosensory system. As well as a reduced beta rebound amplitude, there is also evidence of delayed or prolonged rebound in clinical populations (e.g., Proudfoot et al., 2017). We examined whether similar deviations could be seen in relation to aging, but we observed no between-group differences (latency data for the peak suppression/rebound not reported). The older adults in the present study were healthy and did not show symptoms of neuronal degeneration, thus, it seems that healthy aging is not accompanied with changes in beta rebound amplitude or latency of suppression/rebound.

It is thought that beta suppression and rebound are GABA-mediated processes. Since dichotomous responses can occur between these phenomena (Hall et al., 2011) and since some studies have observed differing locations of peak suppression and rebound (Parkkonen et al., 2015; Bardouille and Bailey, 2019), it has been proposed that suppression is caused by GABA_A_ while rebound is due to GABA_B_-related processes (Muthukumaraswamy et al., 2013). Diazepam is a non-selective GABA_A_ agonist, which increases both resting beta power and post-stimulus beta suppression (Muthukumaraswamy et al., 2013). Since beta rebound is not affected by diazepam administration (Hall et al., 2011), it seems likely that other mechanisms govern the amplitude of beta rebound. Second, and in support of the proposal that different mechanisms govern beta responses, there have been some observations that beta suppression and rebound are generated in different brain regions (Jurkiewicz et al., 2006; Muthukumaraswamy et al., 2013): SM1 and M1, respectively. One small weakness of the present study was that, without structural MRIs, accurate source location was not possible and could not confirm previous findings of different source locations for suppression and rebound. In the future, it may be of interest to manipulate GABAergic neurotransmission (e.g., pharmacologically) to determine whether this influences: (1) stretch-reflex induced beta dynamics; and (2) perturbed balance control.

### Strength of Beta Power at Rest

Resting beta is thought to be indicative of the level of ongoing inhibition, and pre-stimulus power at ~10, ~20, and ~40 Hz above the sensorimotor cortex seems to predict detection of tactile stimulation (Linkenkaer-Hansen et al., 2004). Rather than being genuinely oscillatory, it has been proposed that beta power is a consequence of a series of “events” of specific neuronal activation that have an inhibitory effect for forthcoming sensory input (Jones et al., 2007; Shin et al., 2017). It is possible that the functional consequences of high beta power extend to sensorimotor function, and that the requirement to overcome such inhibition prior to optimal motor command may influence standing balance. For example, it could be hypothesized that high beta power or the close proximity of a beta “event” prior to perturbation would be reflected in an unsuccessful recovery of balance, and this should be elucidated in future research.

### Relationship to Dynamic Standing Balance Performance

The older subjects in the present study were healthy and had good physical functioning, as evidenced by the limited significant between-group differences in recovery of perturbed standing balance. However, the observed relationships between stronger beta suppression and worse balance performance suggests that cortical sensorimotor processing has functional relevance even in the case of rapid postural perturbations. To our knowledge, the only other study evaluating the effects of passive ankle rotations was conducted by Toledo et al. (2016). Here, slow (0.5°·s^−1^) rotations that resulted in a shorter beta suppression latency were also sensed earlier (assessed by a button press task). This would have clear implications for overcoming perturbation during standing. It is, however, difficult to compare directly between the studies, since our rotations were fast, stretch-reflex inducing dorsiflexion actions. Since there were no between-group differences in peak suppression latency we cannot confirm that the beta responses in the older subjects were slower *per se*. Also, recovery of perturbed balance requires integration of several sensorimotor networks with e.g., monosynaptic (spinal) short-latency reflexes playing an important role (Piirainen et al., 2013). Such integration and summation of different mechanisms to overall balance recovery may explain the moderate strength of the relationship observed in the present study. Nevertheless, the present study’s data seem to support the notion that cortical proprioceptive processing is sub-optimal in older adults and this may have functional implications for balance.

## Conclusion

In conclusion, the present study observed greater 15–30 Hz (beta) band power prior to rapid ankle rotations (i.e., proprioceptive stimulation), and greater beta suppression post-proprioceptive stimulation in healthy older compared to young adults. Such age-related adaptations may impair cortical control of prompt motor action, such as regaining perturbed balance. Beta suppression following proprioceptive stimulation was related to dynamic balance performance, and so it seems indicative of poorer sensorimotor functioning in older adults.

## Data Availability Statement

The datasets generated for this study are available on request to the corresponding author.

## Ethics Statement

The studies involving human participants were reviewed and approved by University of Jyväskylä Ethical Committee. The participants provided their written informed consent to participate in this study.

## Author Contributions

SW, IT, and HP conceived the study idea. All authors planned the study. SW, SM, and TP acquired ethical approval. SW, JP, SM, and HP collected the data. SW, SM, and JP analyzed the data. All authors revised and approved the manuscript.

## Conflict of Interest

The authors declare that the research was conducted in the absence of any commercial or financial relationships that could be construed as a potential conflict of interest.
